# Semi-Annual Meeting of the New York State Medical Society

**Published:** 1852-08

**Authors:** 


*Semi-Annual Meeting of the New York State Medical Society.*—We copy the following from the New York Medical Gazette:—

The semi-annual meeting of this body was held in the Hall of the Crosby-street College, on Tuesday, 29th June. Professor Clark presided, and read the usual address, which was an able and judicious performance. As it will no doubt be published, we refrain from any comment till it appears. Dr. Cock was appointed secretary, *pro tem.*

Invitations were then read, inviting the Society to visit our various public institutions, and proffering private hospitality at the residences of Drs. Cheesman, Mott, and Stevens, all of which were accepted.

By a liberal usage of this Society, all the physicians present were made honorary members of the State Society for the present session, and privileged to take part in its deliberations.

The attendance was large, and the meetings for business, as adjourned from day to day, were highly interesting.

Dr. Tuthill read a paper on the Statistics of Births, Marriages, and Deaths, which merits high commendation, for its thorough analysis of the objections made to the law, and their refutation.

Professor Lee followed on the causes of the circulation of fluids in vegetables and animals.

Dr. Swett, on Bright's disease of the kidneys, as the next paper, and was followed with remarks by Professor Clark, and illustrations with his powerful microscopes, during a recess.

A discussion on the varied forms of Quackery and the duty of the Society, on motion of Dr. Cash, of Orange county, brought forth remarks from a large number of the members, which concluded the business of the first day.

In the evening the members partook of the elegant hospitality of Dr. Cheesman, in Broadway.

The second day was opened by Dr. Spencer's long and ingenious paper, on his theory of Vegeto-Animal Heat and Life, which he illustrated by colored diagrams, and upon which topic he has evidently bestowed much labor. Of its merits we are not prepared to speak at present, nor until its publication.

The rest of the day was taken up in visiting the Asylums of the Deaf and Dumb, the Blind, Croton water works, &c., until the evening, when the hospitable mansion of Dr. Mott, at Bloomingdale, welcomed the Society to agreeable recreation and refreshment, under the roof of this "Napoleon of Surgery." After which they attended the Pathological Society.

The third day was chiefly spent in visiting the Alms House Department at Bellevue, Blackwell's Island, and Randall's Island, terminating the day by a feast at Dr. Stevens's country seat in Astoria.

The fourth day was devoted to varied business, a paper on the Statistics of Surgery, by Prof. Van Buren, another by Dr. Peet, on the instruction of Deaf Mutes, &c., when the Society adjourned.

The announced report on the experimental treatment of Puerperal Fever, though ordered to be the special business of this semi-annual meeting, was passed over, although several gentlemen were present from day to day prepared to report papers, and for the discussion had it taken place.

The daily papers generally published brief and correct notices of the proceedings of the Society, and this semi-annual meeting may be regarded as highly satisfactory and useful, so that it will probably be repeated next year. Many gentlemen from different and distant parts of the state remained in attendance throughout the entire session.

